# Supplementary material for: Shadow enhancers mediate trade-offs between transcriptional noise and fidelity
Source: PLoS Comput Biol. 2023 May 19;19(5):e1011071. doi: 10.1371/journal.pcbi.1011071 (PMC10234526; doi:10.1371/journal.pcbi.1011071)
Supplement: Attachment — Submitted filename: revision cover letter.docx [file pcbi.1011071.s008.docx]

Dear PLoS Computational Biology, dear reviewers,

We thank you for your quick review of our revision and want to send you an updated manuscript incorporating the suggested minor revisions:

Reviewer #1: The authors have addressed my major concerns about the manuscript. I have three minor comments:
1) Line 239: I assume the authors mean "the noise does NOT change" (written: "the noise does change")

A: We have fixed this typo, thank you for pointing out

2) Fig 3C: Should the x-axes be tau_1, tau_2 rather than T_1, T_2?

A: Thank you - we have replaced the x-axes per the reviewer's suggestion, in Figures 2C, 3C, 4C and a supplementary figure

3) Line 448: There seem to be a couple exceptions to the "broadly consistent" trend in the superadditive case (e.g., the green curves in Fig S.6C, left and the pink curves in Fig S.6C, center/right reverse or at least do not follow the trend from the original parameter set). Given the expected audience of this publication, I think it is important to be explicit that it is possible to find parameter sets for which the observed trends do not hold, even if the authors do not expect these cases to occur in most biologically relevant situations.

A: This makes sense, and we have included in page 22, line 449: "Regarding Figure S.6, notice that despite the general trends, it is still possible to find parameter sets for which the trends do not hold." Such a comment as suggested by the reviewer not only describes the situation, but clarifies that a rigorous mathematical proof would not be possible, thus validating the computational approach.

Reviewer #2: The authors have replied to all my questions and done substantial changes. I am happy to recommend the manuscript for publication in PLoS Computational Biology.

A: We thank the reviewer for their input and their suggestion to publish the manuscript.
